# Genomic and Comparative Transcriptomic Analyses Reveal Key Genes Associated with the Biosynthesis Regulation of Okaramine B in *Penicillium daleae* NBP-49626

**DOI:** 10.3390/ijms25041965

**Published:** 2024-02-06

**Authors:** Yueying Wang, Ling Chen, Wei Fang, Zhen Zeng, Zhaoyuan Wu, Fang Liu, Xiaoyan Liu, Yan Gong, Lei Zhu, Kaimei Wang

**Affiliations:** National Biopesticide Engineering Technology Research Centre, Hubei Biopesticide Engineering Research Centre, Key Laboratory of Microbial Pesticides, Ministry of Agriculture and Rural Affairs, Hubei Academy of Agricultural Sciences, Wuhan 430064, China; yueying.wang@nberc.com (Y.W.); chenling@nberc.com (L.C.); wei.fang@nberc.com (W.F.); cindy68garry@outlook.com (Z.Z.); zhaoyuan.wu@nberc.com (Z.W.); liufang@nberc.com (F.L.); liuxiaoyan@nberc.com (X.L.); gongyan@nberc.com (Y.G.)

**Keywords:** Okaramine B, yield improvement, *Penicillium daleae*, complete genome, transcriptome, novel regulators, tryptophan metabolism

## Abstract

Restricted production of fungal secondary metabolites hinders the ability to conduct comprehensive research and development of novel biopesticides. Okaramine B from *Penicillium* demonstrates remarkable insecticidal efficacy; however, its biosynthetic yield is low, and its regulatory mechanism remains unknown. The present study found that the yield difference was influenced by fermentation modes in okaramine-producing strains and performed genomic and comparative transcriptome analysis of *P. daleae* strain NBP-49626, which exhibits significant features. The NBP-49626 genome is 37.4 Mb, and it encodes 10,131 protein-encoding genes. Up to 5097 differentially expressed genes (DEGs) were identified during the submerged and semi-solid fermentation processes. The *oka* gene cluster, lacking regulatory and transport genes, displayed distinct transcriptional patterns in response to the fermentation modes and yield of Okaramine B. Although transcription trends of most known global regulatory genes are inconsistent with those of *oka*, this study identified five potential regulatory genes, including two novel Zn(II)2Cys6 transcription factors, Reg2 and Reg19. A significant correlation was also observed between tryptophan metabolism and Okaramine B yields. In addition, several transporter genes were identified as DEGs. These results were confirmed using real-time quantitative PCR. This study provides comprehensive information regarding the regulatory mechanism of Okaramine B biosynthesis in *Penicillium* and is critical to the further yield improvement for the development of insecticides.

## 1. Introduction

Agricultural pests are highly prevalent and have the potential to lead to decreases in production of over 30% in severe cases, resulting in annual economic losses of billions of dollars [1,2]. Various strategies have been employed to prevent, reduce, or manage pests in economically important crops, with pesticide application being the predominant and most pervasive method [1]. The development of resistance in a diverse array of pests has resulted from the frequent and indiscriminate application of chemical insecticides in large quantities and excessive dosages [3]. As public concern regarding environmental ecology, health, and safety has increased, numerous high-risk chemical insecticides have been restricted or banned. The development of low-risk, high-efficiency insecticides derived from natural sources has garnered significant interest and progress in recent years [4,5].

*Penicillium* produces a wide variety of secondary metabolites that have been found to exhibit anti-bacterial, anti-cancer, anti-fungal, anti-inflammatory, and anti-insect properties, with possible applications in the control of invertebrate pests important in agriculture, animal and human health, and other fields [5,6]. Indole alkaloid okaramines were initially discovered and characterized in *Penicillium* (Okaramines A–G [7,8,9,10] and N–R [11]). The congeners were subsequently isolated and identified in *Aspergillus* (Okaramines H–I [12] and S–U [13]) and *Chrysosporium* (Okaramine D, H–K, R and Chrokaramine A–E [14]). Okaramines A–D, G, and Q displayed various levels of insecticidal efficiency, with Okaramine B exhibiting the highest level (approximately 0.2 μg/g LD_50_) [15,16,17]. A previous study demonstrated that the insecticidal activity of Okaramine B occurs mainly through its interaction with the L-glutamate-gated chloride channel (GluCl), which is expressed abundantly in the insect central nervous system. Additionally, it has a specific preference for insects over humans on ligand-gated anion channel selectivity, making it serve as the lead for a new generation of compounds for use in crop protection [18,19].

Moreover, in addition to activity, efficient synthesis plays a crucial role in determining the suitability of a molecule for further insecticide development. The extraction yield of Okaramine B from fermentation is limited to 11.2 mg per kilogram substance [17], whereas Okaramine A yields 15.3 mg [20]. However, the limited production of these compounds poses a challenge for further studies. The core biosynthetic gene cluster (*oka*) responsible for the production of Okaramine B in both *Penicillium* and *Aspergillus* species has been reported to be comprised of the non-ribosomal peptide synthetase gene *okaA*, the flavin-dependent monooxygenase gene *okaB*, the dimethylallyltransferase gene *okaC*, the P450 monoxygenase gene *okaD*, and the α-ketoglutarate-dependent dioxygenase gene *okaE*. Furthermore, the methyltransferase gene *okaF* and an additional P450 monooxygenase gene *okaG* have been detected in *P. ochrochloron* strain ATCC 90288 (formerly AK-40), which has been re-classified from *P. simplicissimum* to *P. ochrochloron* (https://www.atcc.org/products/90288#product-references, accessed on 2 February 2024) [21,22]. In contrast to the alternative gene clusters associated with secondary metabolite synthesis, the *oka* gene cluster did not contain or flank any regulatory or transport-related genes. In addition, the regulatory mechanisms of okaramine biosynthesis remain unknown. Artificial synthesis of okaramines is an alternative and efficient method, and there have been reports of the concise total synthesis of Okaramine C [23], M [24], N [25], J, L, and S–U [26]. However, the synthesis of Okaramine A, B, or its congeners with superior insecticidal properties has not yet been documented.

In the present study, we observed notable variations in the yield of Okaramine B within the same fungal strain when subjected to different fermentation conditions. Consequently, genomic and comparative transcriptomic analyses were conducted on *P. daleae* NBP-49626 between the two fermentation modes to identify the pertinent genes responsible for the regulation, metabolism, and transport of Okaramine B, which exhibited a significant yield difference. The investigation of differentially expressed genes (DEGs) of *P. daleae* NBP-49626 revealed discernible transcriptional patterns of the *oka* gene cluster across the two fermentation modes and identified genes involved in the regulation and transport of Okaramine B biosynthesis. Additionally, the metabolic pathway from tryptophan to formyl-anthranilate may play a role in Okaramine B production. This study offers valuable insights into the mechanisms underlying the regulation of Okaramine B biosynthesis.

## 2. Results

### 2.1. The Yield Differences of Okaramine B

**Figure 1:**
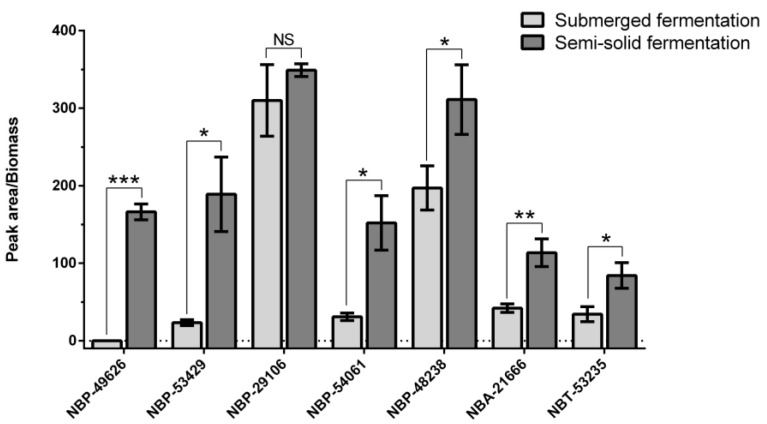
Determination of Okaramine B production of fungal strains in both submerged and semi-solid fermentations for three days. The level of significance was established at *p* < 0.05 (labeled as *), *p* < 0.01 (labeled as **), and *p* < 0.001 (labeled as ***). NS indicates no significant difference.

In this study, seven fungal strains were found to exhibit a significant capacity of Okaramine B production after a short fermentation process, including *Penicillium* sp. strains NBP-49626, NBP-53429, NBP-29106, NBP-54061, and NBP-48238, *Aspergillus* sp. strain NBA-21666, and *Talaromyces* sp. strain NBT-53235. Furthermore, Okaramine B production (Figure 1) showed that six of the seven fungal strains showed varying degrees of yield differences between liquid and semi-solid fermentation on the same medium within a three-day period. Strain NBP-49626 showed significant biomass yield during semi-solid fermentation, whereas Okaramine B was not detected in the liquid broth. The other five strains also produced larger yields in semi-solid fermentation, with yield differences ranging from 1.6 to 8.1 times greater than those in liquid broth (Figure 1).

**Figure 2:**
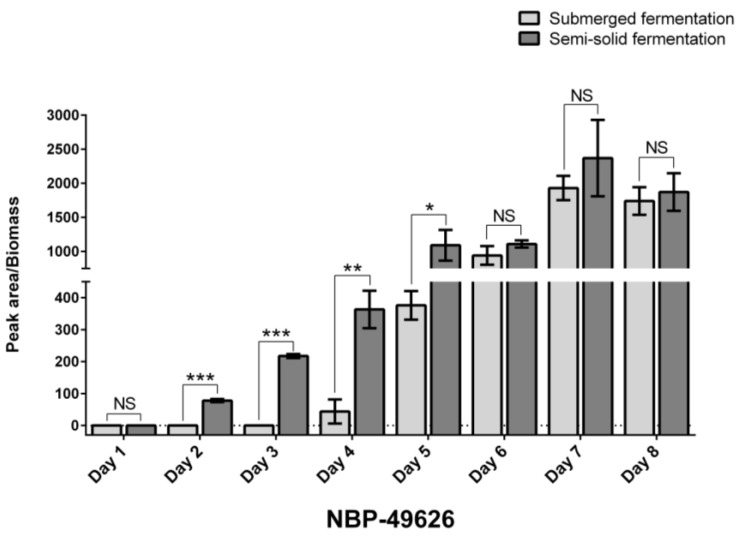
Sequential determination of Okaramine B production of *Penicillium daleae* NBP-49626 in both submerged and semi-solid fermentations. The level of significance was established at *p* < 0.05 (labeled as *), *p* < 0.01 (labeled as **), and *p* < 0.001 (labeled as ***). NS indicates no significant difference.

Given the significant difference in Okaramine B yield, an assessment of the Okaramine B biosynthesis process, as well as an analysis of genome and transcriptome sequencing data, was performed on strain NBP-49626 (using sequential time-point tracking sampling under the two culture conditions) to elucidate the genes responsible for Okaramine B biosynthesis and the regulatory mechanisms involved. Sequential sampling and Okaramine B detection revealed no significant difference in the biomass (wet weight) of the organisms between submerged and semi-solid fermentation throughout an eight-day period; however, strain NBP-49626 exhibited limited synthesis of Okaramine B in substantial quantities during the initial three days of submerged fermentation. In contrast, an evident increase in Okaramine B biosynthesis was observed from the second day of semi-solid fermentation. By day 6 to 8, the yields achieved by both fermentation processes were comparable (Figure 2).

### 2.2. Genomic Data Evaluation

ONT sequencing of the NBP-49626 genome generated 387,760 filtered long reads (7,926,827,031 bp), with a mean length of 20,442.61 bp and an N50 length of 29,025 bp. MGI sequencing produced 23,614,314 filtered short reads (3,542,147,100 bp), obtained from the 200–400 bp paired-end DNA library.

As presented in Table 1, the consensus assembly revealed that the total length of the NBP-49626 genome was 37,366,436 bp, and it consists of eight chromosomes with an N50 length of 4,967,589 bp, resulting in 212.1-fold coverage on ONT and 94.8-fold coverage on MGI. The G + C content of the genome was 49.07%. A total of 10,131 protein-coding genes were predicted in the NBP-49626 genome. The average size of the protein-coding genes is 1663.62 bp, with an average of 3.24 exons per gene. Among the ncRNAs, 58 rRNAs (9, 10, and 39 copies of 18S, 28S, and 5S rRNAs, respectively), 164 tRNAs, and 36 sRNAs were identified. Based on public databases, 9991 of 10,131 genes (98.62%) were successfully annotated. Of these genes, 9983 (98.54%), 7676 (75.77%), 6025 (59.47%), 3514 (34.69%), 3347 (33.04%), 2771 (27.35%), 1435 (14.16%), and 177 (1.74%) were annotated using the NCBI NR, SWISS-PROT, GO, KEGG, KOG, PHI-base, CAZy, and Fungal cytochrome P450 databases, respectively. And 715 (7.06%) secreted protein genes were identified using a combination of signal peptide and transmembrane domain prediction (Table S1). In addition, the phylogenetic analysis based on the concatenated sequence alignments of the molecular identification markers (including rDNA internal transcribed spacer (*ITS*), β-tubulin (*BenA*), calmodulin (*CaM*), and RNA polymerase II second-largest subunit regions (*RPB2*) of strain NBP-49626 and closely related *Penicillium* type strains) indicates that it belongs to *Penicillium daleae* (Figure S2).

**Figure 3:**
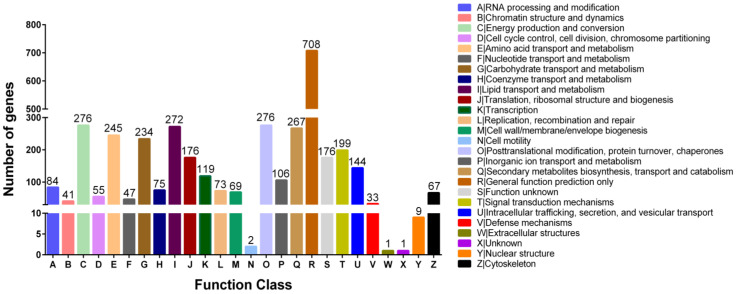
COG function classification of the *Penicillium daleae* NBP-49626 genome. The number on the top of the categories represents the number of genes.

Using NCBI COG mapping, 3755 genes were assigned to COG categories. “General function prediction only” had the highest number of genes, followed by “Energy production and conversion”, “Posttranslational modification, protein turnover, chaperones”, “Lipid transport and metabolism”, “Secondary metabolites biosynthesis, transport and catabolism”, “Amino acid transport and metabolism”, “Carbohydrate transport and metabolism”, “Signal transduction mechanisms”, “Translation, ribosomal structure and biogenesis”, and “Function unknown” (Figure 3).

### 2.3. Transcriptomic Data Evaluation

RNA-seq analysis of 45 samples collected over the course of eight days under the two culture conditions of *P. daleae* strain NBP-49626 generated 141,631,730,858 bp (141.6 Gb) sequence data, consisting of 944,674,938 filtered reads. The mean quality of high-quality sequences (Fastq QC > 30) following adapter trimming was 92.3 ± 0.3%. Approximately 96.59 ± 0.01% of the clean reads were mapped to the *P. daleae* strain NBP-49626 genome, indicating that the quality of transcriptome sequencing data was reliable (Table S2).

**Figure 4:**
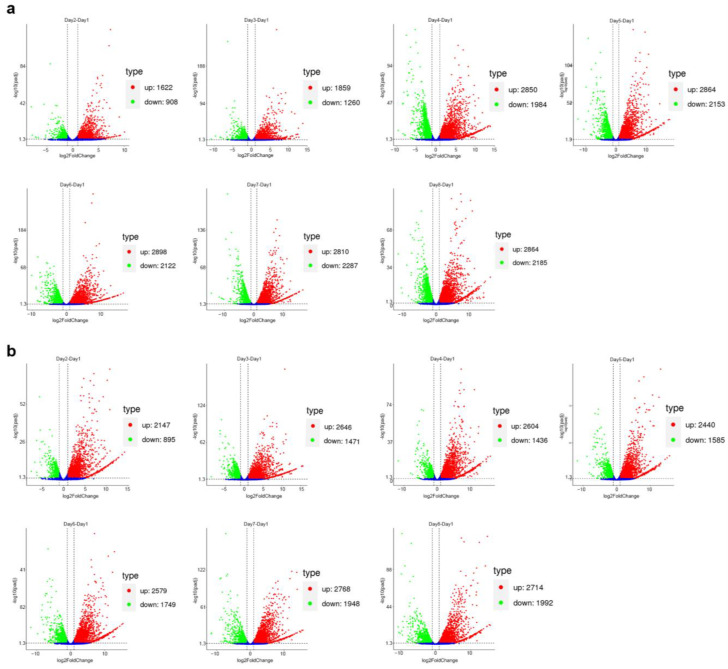
Volcano plots of the pairwise comparisons of various periods during the submerged fermentation (**a**) and semi-solid fermentation (**b**) processes. The differentially expressed genes (DEGs) in six comparison groups are shown as red (up-regulation) and green (down-regulation) spots, and genes that had no significant changes are denoted in blue.

The transcriptome profiles exhibited significant changes at different time intervals throughout the submerged and semi-solid fermentations. A total of 2530 DEGs (fold change ≥ 2.0, *p*-value ≤ 0.05) were identified at the second day of submerged fermentation in comparison to the transcriptome level on the first day, with the number of DEGs essentially stabilizing at high levels from the fifth day onwards (5017 DEGs, Figure 4a, Tables S3 and S4); in contrast, the number of DEGs eventually stabilized at high levels during semi-solid fermentation beginning on days two and three (3042 and 4117 DEGs, respectively; Figure 4b, Tables S3 and S4). The temporal patterns in the varying levels of gene transcription were generally consistent with the trends in Okaramine B yield observed during the corresponding fermentations described above (Figure 2).

**Figure 5:**
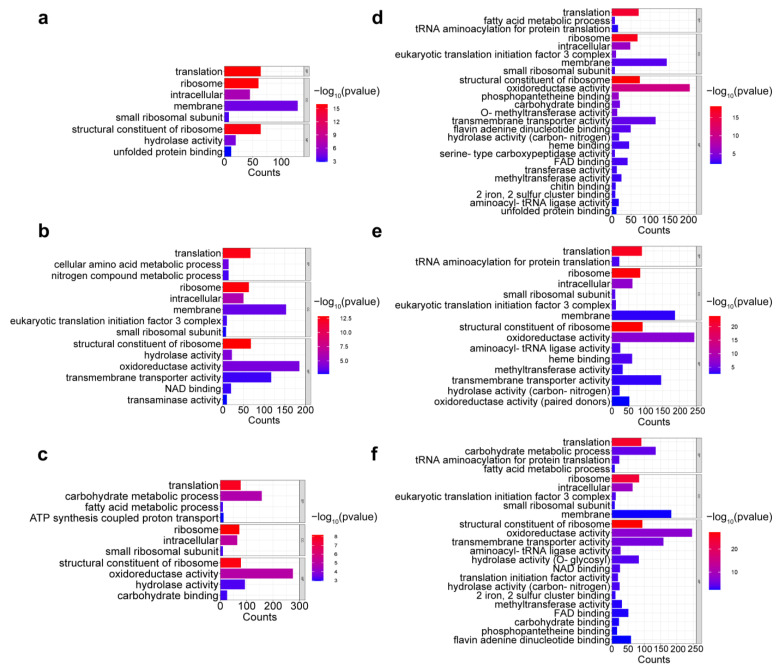
Gene Ontology (GO) functional annotation and classification of differentially expressed genes (≥2-fold change) at various periods during the fermentation processes. (**a**–**c**) Days 2, 3, and 4 vs. day 1 in submerged fermentation. (**d**–**f**) Days 2, 3, and 4 vs. day 1 in semi-solid fermentation.

**Figure 6:**
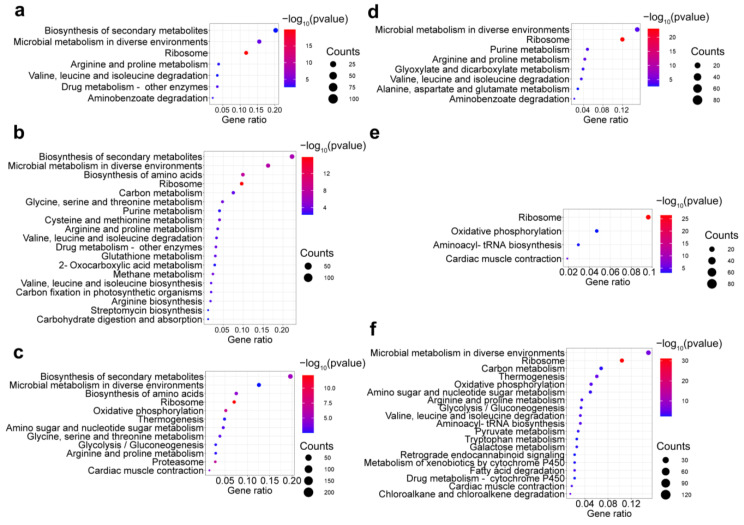
KEGG pathway enrichment analysis of differentially expressed genes (≥2-fold change) at various periods during the fermentation processes. (**a**–**c**) Days 2, 3, and 4 vs. day 1 in submerged fermentation. (**d**–**f**) Days 2, 3, and 4 vs. day 1 in semi-solid fermentation. GeneRatio = DEGs number/total gene number identified from transcriptome of a certain process.

GO term analysis was conducted to identify the biological processes, molecular functions, and cellular components that contribute to Okaramine B production. It revealed that during the submerged fermentation process, the DEGs were primarily associated with molecular functions including oxidoreductase activity (e.g., 185 genes on day three and 275 genes on day four) and transmembrane transporter activity on the third day (117 genes), biological processes including translation (e.g., 67 genes on day three and 77 genes on day four) and carbohydrate metabolic processes between the fourth and sixth days (157, 154, and 152 genes, respectively), and cellular component terms such as membrane between the second and third days (129 and 153 genes, respectively), ribosome (e.g., 63 genes on day three and 72 genes on day four), and intracellular (e.g., 50 genes on day three and 64 genes on day four) (Figure 5a–c and Figure S3). During the semi-solid fermentation process, the DEGs were associated with molecular functions including oxidoreductase activity and transmembrane transporter activity virtually at all times (e.g., 203 genes and 244 genes on day two, respectively), biological processes including translation (e.g., 70 genes on day two), and especially the carbohydrate metabolic processes started on day four (135 genes), as well as cellular component terms including membrane (e.g., 143 genes on day two), ribosome (e.g., 67 genes on day two), and intracellular (e.g., 48 genes on day two). Notably, in semi-solid fermentation, DEGs involved in oxidoreductase activity and transmembrane transporter activity remained for a longer amount of time than those in submerged fermentation (Figure 5d–f and Figure S3). KEGG analyses further revealed these DEGs to be enriched in the biosynthesis of secondary metabolites (map01110, e.g., 148 genes on day three), microbial metabolism in diverse environments (map01120, e.g., 108 genes on day three), and the biosynthesis of amino acids (map01230, e.g., 80 genes on day four) and ribosomes (map03010, e.g., 75 genes on day four) during the submerged fermentation process (Figure 6a–c and Figure S4). On the other hand, these DEGs were mainly enriched in microbial metabolism in diverse environments (e.g., 84, 121, and 123 genes on days two, four, and six, respectively) during the semi-solid fermentation process (Figure 6d–f and Figure S4).

### 2.4. Differential Expression of Okaramine Biosynthesis Gene Cluster

Homologs of the *oka* gene cluster have been identified in the genome of *P. daleae* NBP-49626. The amino acid (aa) identities of the proteins encoded by these homologs (OkaA to OkaG) were 96.84%, 98.67%, 97.44%, 98.49%, 99.00%, 98.95%, and 99.24%, respectively.

**Figure 7:**
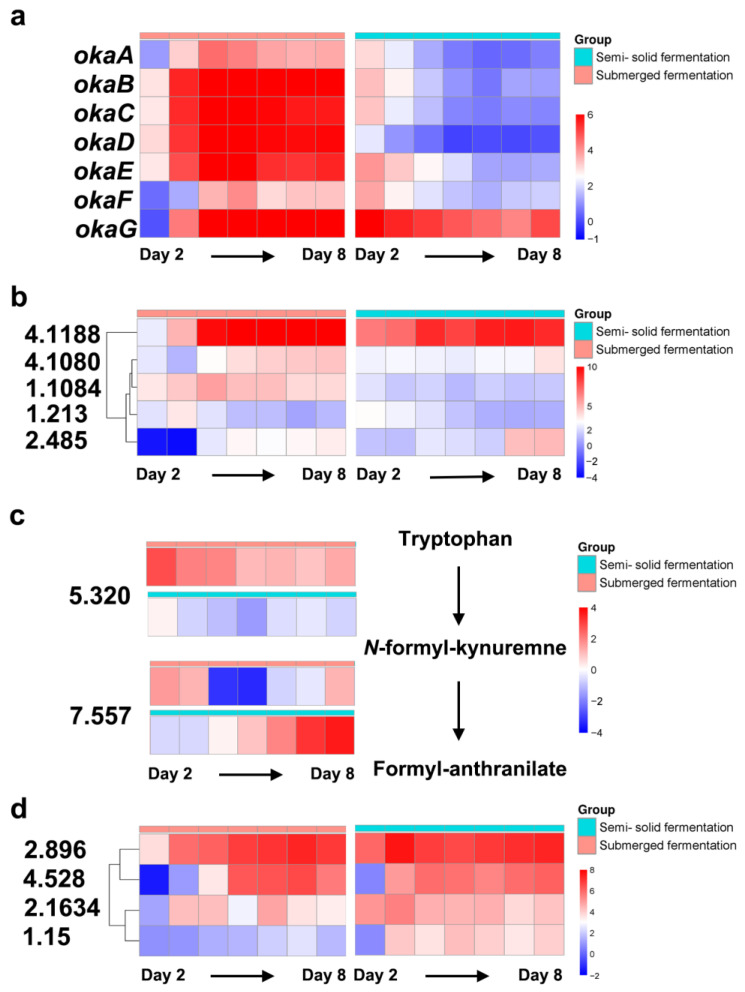
Expression patterns of key genes involved in Okaramine B biosynthesis at various periods during the fermentation processes. (**a**) The *oka* gene cluster of Okaramine B biosynthesis. (**b**) Putative regulator genes. (**c**) Genes involved in tryptophan metabolic pathway. (**d**) Putative transporter genes. The blue to red color scale values represent fold changes.

The *oka* gene cluster exhibited a notable gradual increase in up-regulation from the second to the third day of submerged fermentation (average 4.3-fold up-regulated of these seven genes), and this level of up-regulation of gene transcripts was sustained until the eighth day (average 5.5-fold up-regulated, Figure 7a, Table S5), mirroring the trend observed in the yield enhancement of Okaramine B (Figure 2). Contrariwise, in semi-solid fermentation, the production of Okaramine B increased from the first to the second day (Figure 2), and the levels of *oka* gene cluster transcripts significantly increased by an average of 3.8-fold from the second day onwards but decreased relative to the later stages (average 1.6-fold on day eight; Figure 7a, Table S5).

During the biosynthesis of Okaramine B, the protein products encoded by the *oka* gene clusters exhibit their roles sequentially, with OkaA, OkaC, OkaB, OkaD, OkaE, OkaG, and OkaF [21,27]. More detailed analyses revealed that the differences in the patterns of the two transcript levels suggested two distinct synthesis strategies. In contrast to submerged fermentation, in which the transcript levels of all seven genes increased with fungal growth, the transcript levels of genes involved in the initial steps of Okaramine B synthesis were significantly higher on the third day of semi-solid fermentation, resulting in a greater peak of Okaramine B production. During semi-solid fermentation, the expression levels of the *okaA*, *okaC*, *okaB*, and *okaD* genes involved in the initial synthesis of Okaramine B decreased from the third to the fourth day (e.g., 3.1-fold on day two and 1.4-fold on day four of gene *okaA*, respectively), although the production of Okaramine B was still at its highest point (Figure 2). In contrast, the *okaE* and *okaF* genes responsible for the subsequent steps showed a slower increase and then a decrease in expression levels (e.g., 4.0-fold on day two and 2.7-fold on day four of gene *okaE*, respectively). Notably, the *okaG* gene remained consistently up-regulated at a high level (e.g., 6.4-fold on day two and 5.0-fold on day eight, respectively; Figure 7a, Table S5).

### 2.5. Identification of the Differentially Expressed Regulator Genes

Given the lack of regulatory-related genes in the *oka* gene cluster [21], the control of Okaramine B synthesis may be derived from regulatory factors elsewhere in the genome. In accordance with the genome annotation data, 259 regulatory genes were identified among the DEGs. Investigation of the transcription patterns of these regulatory genes, combined with the patterns of the *oka* gene clusters under the two fermentation processes, suggested that five regulatory genes, including genes 1.1084, 1.213, 2.485, 4.1080, and 4.1188, could play a role in the regulation of Okaramine B biosynthesis (Figure 7b).

The transcription trend of gene 1.1084 exhibited a similar pattern to that of the Okaramine B biosynthesis gene cluster during submerged fermentation (e.g., 3.7-fold on day two, 5.8-fold on day four, and 4.1-fold on day eight, respectively; Figure 7b, Table S5). Although the level of up-regulation was statistically significant during semi-solid fermentation, it was lower than that observed during submerged fermentation (e.g., 2.4-fold on day two, 1.8-fold on day four, and 1.5-fold on day eight, respectively; Figure 7b, Table S5). The annotation results derived from the NCBI NR database indicated that the protein Reg2, encoded by gene 1.1084, shares 87.7% aa identity with the transcriptional regulatory protein pro-1 found in *P. brasilianum* (accession no. OOQ84656.1). Conserved domain analysis revealed that Reg2 belongs to the Zn(II)2Cys6 transcription factors family (domain architecture ID 10049156), which includes a GAL4-like Zn2Cys6 binuclear cluster DNA-binding domain (GAL4; accession no. cd00067) and a fungal-specific transcription factor domain (Fungal_trans_2; accession no. pfam11951). The transcriptional pattern of gene 4.1080 during submerged fermentation was similar to that of gene 1.1084. However, in the latter stages of the semi-solid fermentation process, gene 4.1080 did not exhibit a decrease in transcriptional up-regulation as Okaramine B production stabilized. Instead, it maintained a consistently high level of up-regulation (3.8-fold on day eight) (Figure 7b, Table S5). Protein Reg21, encoded by gene 4.1080, shares 76.9% aa identity with the conidiophore development regulator BrlA in *P. rubens* (accession no. B6GVZ2.1) [28]. It was hypothesized that unlike gene 4.1080, gene 1.1084 might be associated with the regulation of Okaramine B biosynthesis during submerged fermentation.

Gene 1.213 exhibited significant transcriptional activity during the initial stages of both liquid (2.3-fold to 3.7-fold between day two and four) and semi-solid fermentation conditions (3.2-fold to 2.3-fold between day two and four; Figure 7b, Table S5). Protein Reg19, encoded by gene 1.213, shares 91.5% aa identity with the Zn(II)2Cys6 transcription factor in *P. brasilianum* (accession no. OOQ91732.1). The transcription factor belongs to another group of Zn(II)2Cys6 transcription factors (domain architecture ID 10048070) and contains a domain of fungal transcription factor regulatory middle homology region (fungal_TF_MHR; accession no. cd12148) and a GAL4 domain. It is postulated that Reg19 may play a role in promoting the initiation of Okaramine B biosynthesis.

The expression levels of genes 2.485 and 4.1188 were delayed in relation to Okaramine B biosynthesis during both liquid and semi-solid fermentation conditions (Figure 7b, Table S5). Notably, gene 2.485 displayed down-regulation in transcription at the onset of liquid fermentation, but later transitioned to a significant up-regulation on the fourth day, coinciding with a substantial increase in Okaramine B production. Protein conserved domain and function analysis indicated that the protein Reg5, encoded by gene 2.485, shares 66.9% aa identity with the transcription factor ste11 in *P. subrubescens* (accession no. OKP12827.1) and an HMG-box_ROX1-like domain (accession no. cd01389). Protein Reg8, encoded by gene 4.1188, shares 40.1% aa identity with the actin cortical patch SUR7/pH-response regulator PalI in *P. camemberti* (accession no. CRL27546.1) without any known conserved domains. These two genes are believed to be associated with the repression of Okaramine B biosynthesis.

### 2.6. Identification of the DEG Involved in Tryptophan Metabolism

Tryptophan serves as a substrate for Okaramine B [21]. Sixteen DEGs were found to be involved in the tryptophan metabolism. Comparative analysis of transcript levels on day two revealed that genes 5.320 and 7.557 were up-regulated by 2.8-fold and 1.6-fold, respectively, during submerged fermentation. In contrast, they were down-regulated by 1.6-fold and remained unchanged during semi-solid fermentation. The putative kynureninase encoded by gene 5.320 and indoleamine 2,3-dioxygenase encoded by gene 7.557 are responsible for the metabolic pathway from tryptophan to *N*-formyl-kynuremne and from *N*-formyl-kynuremne to formyl-anthranilate, respectively (Figure 7c). The significant transcriptional differences between the two fermentation processes during the initial stages demonstrated that the pathway from tryptophan to formyl-anthranilate may play a role in regulating Okaramine B production via substrate competition.

Furthermore, during the early phases of the two fermentation processes, genes 3.452 and 3.457, which encode monoamine oxidases, displayed notable distinctions: they remained unaltered during submerged fermentation, and they were up-regulated by 2.3- and 2.2-fold, respectively, during semi-solid fermentation (Table S5). They play important and comprehensive roles in numerous tryptophan metabolic pathways, including those from tryptamine to indole-3-acetaldehyde and from serotonin to 5-hydroxyindole-acetaldehyde.

### 2.7. Identification of the DEG Involved in ATP-Binding Casette (ABC) Transporters

Transmembrane transport also plays an important role in the biosynthesis and accumulation of secondary metabolites [29,30]. A total of 65 genes were identified as ABC transporters among the DEGs. Seventeen of these genes exhibited varying levels of transcriptional up-regulation during both the fermentation processes.

The ABC multidrug transporter gene 1.15 showed significant up-regulation on the third day of semi-solid fermentation (4.1-fold) and was maintained until the eighth day (4.0-fold). In contrast, it was up-regulated from the fourth day of submerged fermentation (1.5-fold) and continued to be up-regulated until the eighth day (1.7-fold). This pattern of transcriptional up-regulation aligns with the biosynthesis of Okaramine B, although the level of up-regulation in submerged fermentation was generally lower than that in semi-solid fermentation. Gene 2.1634 exhibited a considerable increase in expression during both submerged and semi-solid fermentation (1.2-fold and 5.2-fold, respectively). This up-regulation was observed in the early stages and maintained at high levels of transcription, with a 3.4-fold and 4.2-fold increase on day eight, respectively. Gene 2.896 exhibited a large increase in expression levels (3.7-fold and 6.0-fold, respectively) during the initial phases of biosynthesis and maintained high levels of transcription (7.0-fold and 7.3-fold on day eight, respectively). Gene 4.528 had a considerable increase in expression starting from the third day, with fold changes of 1.0 and 5.1, respectively. Furthermore, high levels of transcription were maintained, with fold changes of 5.7 and 6.1 on the eighth day, respectively. Nevertheless, these genes exhibited a slower growth rate during submerged fermentation than during semi-solid fermentation (Figure 7d, Table S5).

### 2.8. Validation of RNA-Seq Results by RT-qPCR

**Figure 8:**
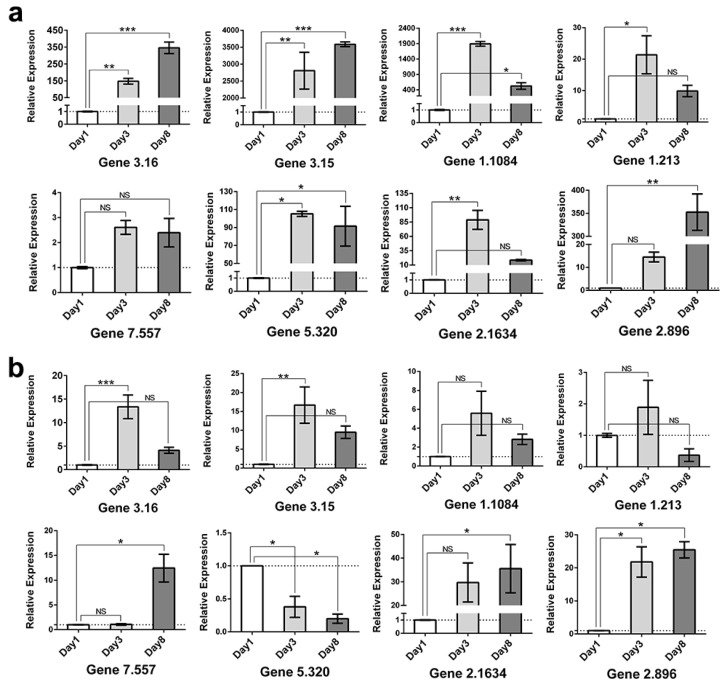
The verification of relative expression levels of genes by RT-qPCR during the submerged fermentation (**a**) and semi-solid fermentation (**b**) processes. Gene 3.16, the non-ribosomal peptide synthetase gene *okaA*; Gene 3.15, the flavin-dependent monooxygenase gene *okaB*; Gene 1.1084, transcriptional regulatory protein Reg2; Gene 1.213, transcriptional regulatory protein Reg19; Gene 7.557, indoleamine 2,3-dioxygenase; Gene 5.320, putative kynureninase; Gene 2.1634, transporter gene; Gene 2.896, transporter gene. The level of significance was established at *p* < 0.05 (labeled as *), *p* < 0.01 (labeled as **), and *p* < 0.001 (labeled as ***). NS indicates no significant difference.

Eight DEGs associated with Okaramine B biosynthesis, regulatory factors, tryptophan metabolism, and ABC transporters were selected for RT-qPCR (Table 2). These results were consistent with the RNA-Seq data (Figure 8), confirming a comparable expression pattern of up- and down-regulated genes. These findings can be utilized for subsequent studies.

## 3. Discussion

Fungi generate a diverse range of bioactive secondary metabolites, which could be associated with their function as decomposers in ecosystems, as well as their role as plant mutualists and nutrient-rich food sources for numerous insects [4,31]. Thus, they have developed the capacity to generate large quantities of compounds that possess insecticidal, alluring, and even carnivorous properties during their long struggle against insects [5,32]. For example, the indole-diterpenoid compound penitrem G, isolated from *P. crustosum*, shows convulsive and insecticidal activities against *Oncopeltus fasciatus* and *Ceratitis capitata* [33]. Pyripyropene A, extracted from *P. coprobium* and *P. reticulisporum*, inhibits acetylcholinesterase and possesses significant insecticidal efficacy against aphids. It serves as a prototype for the development of novel insecticides, including Afidopyropen [34]. The compound 2-furoic acid has nematicidal properties. It effectively poisoned nematodes upon infection and significantly enhances the nematode-capturing ability of *Dactylellina haptotyla* [35]. Despite the discovery of a considerable number of insecticidal metabolites in fungi (e.g., more than 150 compounds in *Penicillium* spp. [5]), only a minority have progressed to the stage of development into insecticides. Additional in-depth research on insecticidal activity, spectrum, environmental safety, and development of insecticide products is significantly impeded by the capacity to manufacture large quantities of naturally or artificially synthesized pesticides. Understanding the biosynthetic gene clusters (BGCs) of secondary metabolites produced by fungi has rendered the transcriptional regulation of such BGCs a crucial factor in elucidating the efficacy of product biosynthesis. A substantial variation in the yield of Okaramine B was observed because of fermentation conducted under distinct culture conditions. Using comparative transcriptomics, this study sought to investigate the transcriptional differences of *oka* cluster and regulatory-related genes.

Approximately 50% of fungal BGCs contain a transcription factor unique to the cluster. This cluster-specific transcription factor is often a C6-zinc cluster protein that can identify palindromic patterns in the promoters of genes inside the cluster [4,36]. In this study, the gene cluster *oka* of Okaramine B did not contain or flank regulatory-related genes [21]. In addition to cluster-specific transcription factors, secondary metabolite regulation also depends on global regulation. The Velvet complex, consisting of LaeA, VeA, and VelB, is the most influential transcriptional complex known to affect the global regulation of secondary metabolites in fungi [37]. In the transcriptome analysis conducted in this study, gene 4.839, encoding the protein with 66.74% aa identity with LaeA in *P. rubens* (accession no. B6H9U8.1), exhibited transcriptional up-regulation starting from the fourth day. However, no significant transcriptional alterations were observed in genes 4.494 or 1.65. These two genes encode proteins that share 75.0% and 72.1% aa identity, respectively, with a sexual development activator VeA in *P. citrinum* (accession no. BAL61195.1) and VelB in *P. rubens* (accession no. B6HU70.1). Similar cases that exhibited non-differential expression included the following: gene 1.2132, the protein of which shares 52.9% aa identity with the global negative regulator McrA of BGCs in *Penicillium* and *Aspergillus* species [38]; gene 1.1524, the protein of which shares 77.1% aa identity with the Zn(II)2-Cys6-type regulator KpeA in *A. oryzae* (accession no. Q2UJJ4.1) [39]; gene 5.211, the protein of which shares 76.9% aa identity with Myb domain-containing transcription factor in *Hapsidospora chrysogena* (accession no. AIZ05820.1) [40]; and so on.

However, it is interesting to note that this study identified, in addition to the homologs of the regulator BrlA (gene 4.1080), some regulatory genes that potentially influence the biosynthesis of Okaramine B. Although the coding gene of the KpeA homolog did not show significant transcriptional differences as described above, it was found that regulators Reg2 and Reg19 (which also belong to different groups of Zn(II)2Cys6 transcription factors) probably perform distinct functions at various stages of Okaramine B production. Cluster-specific Zn(II)2Cys6 transcription factors inside the BGCs, such as MlcR in *P. citrinum* [41] and CgcheR in *Chaetomium globosum* [42], usually act as transcriptional activators in fungi. Investigating whether Reg2 and Reg19 exhibit regulatory effects comparable to those of similar transcriptional regulators inside BGCs will be a crucial focus for future research.

Moreover, a previous study showed that the biosynthesis of pentacyclic triterpenes in *Saccharomyces cerevisiae* was significantly increased by up to 127-fold through the overexpression of the mevalonate pathway and suppression of a competing pathway (sterol synthesis) [43]. This demonstrates that the regulation of metabolic flux direction is another crucial determinant of secondary metabolite production. Tryptophan serves as a building block for several signature indole alkaloids present in fungi. Okaramine B is derived from a combination of two L-tryptophan molecules [21], indicating that tryptophan metabolism likely has a significant influence on the regulation of Okaramine B biosynthesis either directly (e.g., precursor availability) or indirectly (e.g., by affecting gene expression in *oka* cluster). During the semi-solid fermentation process, the conversion of tryptophan to formyl-anthranilate appeared to be inhibited compared to during the submerged fermentation, resulting in different Okaramine B yields. In both processes, the genes responsible for the conversion between tryptophan and anthranilate were down-regulated (e.g., genes 2.442, 4.1036, 2.670, 2.96, and 3.210 in Table S5), along with the Okaramine B biosynthesis. Based on the above results, it is hypothesized that Okaramine B biosynthesis is intricately linked to tryptophan flux in alternative metabolic pathways. In addition, effective transportation of synthetic products is essential for maintaining the continuous and beneficial flow of the biosynthesis pathway [30]. Transporter genes that were up-regulated during Okaramine B production were identified in both fermentation processes. Disruption/deletion mutants of these genes could provide more detailed insights into their roles in the regulation of Okaramine B biosynthesis.

## 4. Materials and Methods

### 4.1. Strains and Culture Conditions

The fungal strains used in this work were *Penicillium* sp. strains NBP-49626, NBP-53429, NBP-29106, NBP-54061, and NBP-48238, *Aspergillus* sp. strain NBA-21666, and *Talaromyces* sp. strain NBT-53235. These strains were cultivated on potato dextrose agar medium (PDA, 200 g/L potato extracts, 20 g/L glucose, and 15 g/L agar) at 28 °C and preserved at 10 °C in darkness.

For spore suspension preparation, one-week-old culture dishes incubated at 28 °C were flooded with sterile distilled water containing 0.02% Tween 80. The concentration of the spore suspension was determined using a blood-counting chamber. Based on the difference in sporulation ability of these strains, the inoculation amount in the following assays was normalized to 5 × 10^6^ spores/mL as the final concentration in a lab-modified medium (6.25 g/L maltose, 6.25 g/L malt extract, 1 g/L yeast extract, 0.625 g/L peptone, 1.25 g/L KH_2_PO_4_, 0.625 g/L MgSO_4_∙7H_2_O, pH 7.0, supplemented with 3 g/L agar or not) for submerged or semi-solid fermentation. Both cultures were grown in 50 mL of media in Erlenmeyer flasks and incubated at 28 °C for one or two weeks, depending on the results of Okaramine B detection. The liquid cultures were shaken at 150 rpm, and semi-solid cultures were kept still. All treatment groups were prepared in triplicates.

### 4.2. Determination of Okaramine B Production and Mycelial Wet Weight

During fermentation, 2 mL of liquid or semi-solid broth were taken out from each flask daily to detect Okaramine B. Five times methanol, vortexed and fully mixed, was added to each culture broth sample, then supernatant was filtered with a 0.22 μm syringe filter, and two microliters were injected into a C18 reverse-phase column on a Waters Acquity Ultra Performance Liquid Chromatography (UPLC) system (Waters Acquity UPLC BEH C18 1.7 μm, 2.1 × 100 mm, 0.45 mL/min). The mobile phases were water (0.2% acetic acid, A) and acetonitrile (0.2% acetic acid, B). The linear elution gradient was: 0–0.2 min, 95%A; 0.2–4.2 min, 95%A–100%B; 4.2–5.2 min, 100%B; 5.2–5.70 min, 100%B–95%A; 5.70–7.00, 95%A for 10 min, at a flow rate of 0.45 mL/min. The detection parameters of Okaramine B using the multiple reaction monitoring (MRM) mode on electrospray ionization mass spectrometry (ESI-MS, Waters XEVO TQD, Milford, MA, USA) were as follows: parent ion, 567; daughter ion, 531, 172; cone, 40 V; collision, 12 V. The spectra and calibration curve are shown in Figure S1. The remaining liquid broth was filtered through a 100-mesh nylon cloth, and the remaining mycelia in the semi-solid broth were scraped and moved to a 100-mesh nylon cloth. The mycelia on the cloth were quickly washed three times with pre-chilled sterile water, screwed to dry, and moved into a centrifuge tube for centrifugation at 8000 rpm for 5 min at 4 °C. After removing residual water, the mycelia were immediately weighed and stored in liquid nitrogen for sequencing. Because the peak area of Okaramine B measured during the fermentation process of most strains is significantly less than the calibration equation’s lower limit, particularly in the first three to five days, the converted mass concentration is insufficiently precise. In this work, the Okaramine B yield was defined as the peak area per gram wet weight.

### 4.3. Genome DNA Isolation and Sequencing

High-quality genomic DNA was extracted from pure cultured mycelial samples of strain NBP-49626 on a PDA plate using the cetyltrimethylammonium bromide method [44]. The integrity and purity were measured using agarose gel electrophoresis and a NanoDrop™ One UV-Vis spectrophotometer (Thermo Fisher Scientific, Lenexa, KS, USA). The rDNA internal transcribed spacer (*ITS*) sequence was amplified and sequenced followed the *Penicillium* identification protocol (primers ITS1: 5′-TCCGTAGGTGAACCTGCGG-3′, and ITS4: 5′-TCCTCCGCTTATTGATATGC-3′) [45]. *ITS* sequences were analyzed against the local UNITE database (https://unite.ut.ee/, accessed on 2 February 2024). Genome sequencing was performed using the ONT PromethION platform (Oxford Nanopore Technologies, Ltd., Oxford, UK) for long-read sequencing, and the DNBSEQ-T7RS platform (MGI Tech Co., Ltd., Shenzhen, China) for genome assembly polishing using short reads (150 nucleotide length reads). Guppy v.3.2.2 (–c dna_r9.4.1_450bps_fast.cfg) [46] was used to perform base calling and adapter sequence removal from the raw long-read sequences.

### 4.4. Genome Assembly and Gene Annotation

The genome was assembled de novo using the filtered long reads by NextDenovo v.2.0 (https://github.com/Nextomics/NextDenovo/, accessed on 2 February 2024; -genome_size 50m -read_type ont). Racon v.1.4.10 (https://github.com/lbcb-sci/racon/, accessed on 2 February 2024) was used for error correction. The assembly was polished using filtered short reads by NextPolish v.1.1.0 [47]. Genome completeness was assessed using Benchmarking Universal Single-Copy Ortholog (BUSCO) v.4.0.3 (-l fungi_odb10 -g genome) [48].

GeMoMa v.1.6.1 [49], GeneMark-ET v.4.0 [50], and Evidence Modeler v.1.1.1 [51] were employed for gene prediction. ncRNAs were predicted by Infernal v.1.1.2 [52] against the RNA families (Rfam) database [53]. tRNAscan-SE v.2.0.5 (--thread 4 -E -I) [54] and RNAmmer v.1.2 (-S euk -m lsu,ssu,tsu -gff) [55] were used for rRNA prediction. The predicted genes were functionally annotated using the Basic Local Alignment Search Tool (BLAST) v.2.7.1 (-e 10^-5^) [56] alignment against the NCBI non-redundant protein (NR) database, the Kyoto Encyclopedia of Gene and Genomes (KEGG) database, the Eukaryotic Orthologous Groups of protein (KOG) database, the Gene Ontology (GO) database, the SWISS-PROT database, Pathogen Host Interactions (PHI-base) [57], the Carbohydrate-Active enZYmes database (CAZy) [58], and the Fungal cytochrome P450 database (P450) [59]. Annotation completeness was assessed using BUSCO v4.0.3 (-l BUSCO_db -m prot) [48]. SignalP v.5.0 [60] and TMHMM v.2.0 [61] were used to predict signal peptides and transmembrane domains to identify secreted proteins with a signal peptide but no transmembrane domain. Parameter settings were maintained at the default values. Protein conserved domain analysis was performed using NCBI CD-search [62]. The β-tubulin (*BenA*), calmodulin (*CaM*), and RNA polymerase II second-largest subunit regions (*RPB2*) were extracted from the genome by the BLAST algorithm against those from the closely related Penicillium-type strains [22]. For phylogenetic analysis, each set of sequences (*ITS*, *BenA*, *CaM*, and *RPB2*) was aligned using the muscle algorithm, and a phylogenetic tree was constructed using the concentrated sequences by the Maximum Likelihood method in MEGA X [63].

### 4.5. RNA Isolation and Sequencing

Eight days of submerged and semi-solid fermentation of *P. daleae* NBP-49626 were selected for transcriptome analysis. Total RNA was extracted from the frozen mycelia samples with TRIzol Reagent^®^ (Invitrogen, Carlsbad, CA, USA) according to the instructions and digested using DNase I for genomic DNA removal. The RNA quality was evaluated with a NanoDrop™ One UV-Vis spectrophotometer (Thermo Fisher Scientific, Lenexa, KS, USA). The messenger RNA (mRNA) was purified from total RNA using a Dynabeads™ mRNA Purification Kit (Cat#61006, Invitrogen, USA), then mixed and cleaved into 200-700 nt with fragmentation buffer with the MGIEasy RNA Library Prep Kit V3.1 (Cat# 1000005276, MGI Tech Co., Ltd., Shenzhen, China). First-strand cDNA was synthesized using the cleaved RNA fragments as templates with random hexamer primers and reverse transcriptase, followed by second-strand cDNA synthesis using DNA Polymerase I and RNase H. The synthesized cDNA was end-repaired, A-tailed, and ligated to sequencing adapters according to the library construction protocol. The cDNA fragments were amplified using PCR and purified using MGIEasy DNA Clean beads (Cat#1000005279, MGI, Shenzhen, China). The library was analyzed using an Agilent Technologies 2100 bioanalyzer (Agilent Technologies, Santa Clara, CA, USA). Double-stranded PCR products were heat-denatured and circularized using the splint oligo sequence in the MGIEasy Circularization Module (Cat#1000005260, MGI, China). Single-strand circular DNA was used as the final library. Qualified libraries were sequenced on the DNBSEQ-T7RS platform (MGI, China), generating 150 bp paired-end reads. Three replicates were employed at all time points, except for the initial day, when only two replicates were utilized, as this represented the early stage of growth with a lower number of organisms. One replicate failed during the RNA extraction on the seventh day of liquid fermentation. A total of 45 replicates were successfully completed for RNA extraction and sequencing during an 8-day period, using two different growth modes.

### 4.6. Analysis of RNA-Seq Data

Raw reads were filtered using fastp v.0.23.4 [64] for read adaptors, artificial reads, and other low-quality reads. FastQC v.0.12.0 [65] was used for the quality control of clean reads. Using HISAT2 v.2.2.1 [66], the clean reads were mapped to the assembled genome of *P. daleae* NBP-49626. Spliced RNA-Seq read alignments were generated using STAR v.2.7.3 [67] and processed into transcripts using StringTie v.1.3.4 [68]. The fragments per kilobase of exon per million reads (FPKM) method was used to calculate and normalize gene expression. Normalized gene expression levels in the submerged and semi-solid fermentation samples of *P. daleae* NBP-49626 on different days were directly compared and collected using StringTie v.1.3.4 [68]. False discovery rate (FDR) control was performed using DESeq2 v. 1.42.0 [69] for differential gene expression analysis. Genes with a fold change ≥ 2.0, *p*-value ≤ 0.05, and FDR ≤ 0.05 were identified as differentially expressed. GO functional enrichment and KEGG pathway enrichment analysis were performed using clusterProfiler v.4.10.0 [70] with *p*-adjust (FDR) < 0.05.

### 4.7. Real-Time Quantitative PCR Validation

Eight DEGs associated with Okaramine B biosynthesis, regulators, ABC transporters, and tryptophan metabolism were selected to confirm the transcriptome data via RT-qPCR. β-actin (Gene ID 4.104) was used as the inner reference. Primers used in this study were designed using Primer3web v.4.1.0 [71]. Total RNA from eight-day submerged and semi-solid fermentation samples was extracted using the FastPure^®^ Plant Total RNA Isolation Kit (Cat#RC401, Vazyme, Nanjing, China) and reverse transcribed to cDNA using the iScript™ gDNA Clear cDNA Synthesis Kit (Cat#172-5035, Bio-Rad, Hercules, CA, USA). The qRT-PCR analysis was performed on a C1000 Touch™ Thermal Cycler system (Bio-Rad, Hercules, CA, USA) using iTaq Universal SYBR^®^ Green Supermix (Cat#1725122, Bio-Rad, Hercules, CA, USA). Three biological replicates were used in each experiment. The relative expression level of the genes was calculated using the 2^−ΔΔCT^ method.

### 4.8. Statistical Analysis

Statistical analyses were conducted using a one-way analysis of variance (ANOVA) with SPSS Statistics v22.0 (IBM, Armonk, NY, USA), followed by Tukey’s HSD tests. The level of significance was established at *p* < 0.05 and *p* < 0.001.

## 5. Conclusions

In conclusion, genomic and comparative transcriptome analyses were conducted on two distinct fermentation processes, focusing on Okaramine B yield in *Penicillium daleae* NBP-49626. The aim of this study was to gain insights into the regulatory mechanisms governing the biosynthesis of this insecticidal component, which holds promise for its potential development as an insecticide. With annotation of the complete sequenced genome, thousands of DEGs were identified in different periods between the two fermentation processes. The analysis of DEGs revealed distinct transcriptional patterns of the *oka* gene cluster in response to varying fermentation conditions and yield of Okaramine B, identified important regulator and transporter genes, and identified transcriptional changes in metabolic genes of the substrate tryptophan. These findings provide a theoretical foundation for understanding the regulation of biosynthesis and yield improvement of Okaramine B, with great potential for application in the development of insecticides.

## Figures and Tables

**Table 1:** Chromosome-level genome features of *Penicillium daleae* NBP-49626.

Genome Features	Value
Genome size (bp)	37,366,436
Number of chromosomes	8
N50 scaffold length (bp)	4,967,589
N90 scaffold length (bp)	2,981,452
Maximum scaffold length (bp)	7,888,045
Minimum scaffold length (bp)	2,295,385
G + C content (%)	49.07
Complete BUSCOs of genome (%)	99.47
Number of protein-coding genes	10,131
Avg gene length (bp)	1663.62
Avg CDS length (bp)	1479.65
Average number of exons per gene	3.24
Average exon length (bp)	456.45
Average intron length (bp)	82.07
rRNAs	58
tRNAs	164
Complete BUSCOs of genes (%)	99.08
Number of annotated genes by NR	9983
Number of annotated genes by SWISS-PROT	7676
Number of annotated genes by GO	6025
Number of annotated genes by KEGG	3514
Number of annotated genes by KOG	3347
Number of annotated genes by PHI-base	2771
Number of annotated genes by CAZy	1435
Number of annotated genes by P450	177
Number of secreted proteins	715

BUSCO, Benchmarking Universal Single-Copy Orthologs; CDS, coding DNA sequence.

**Table 2:** RT-qPCR primers used in this study.

Gene ID	Primer Name	Primer Sequence 5′→3′
4.104	actin-F	ACAGTCCAAGCGTGGTATCC
	actin-R	CACACGGAGCTCGTTGTAGA
3.16	okaA-F	CTTGATCCGAGCGAGGTTAG
	okaA-R	CGGCTATCGAAGCACTTAGG
3.15	okaB-F	CAAACCAACAAGGCCAAAGT
	okaB-R	TATACCCTCGACCCTCGTTG
1.1084	reg2-F	ACCAGATCAGCTCACCCAAA
	reg2-R	CGATCAAAGCCTGGAACGAG
1.213	reg19-F	TCAGCTCACCAAGTTCCAGT
	reg19-R	CTTCTCGATCTCCTCTGCGT
7.557	try7-F	TGATCTCTGGTGCCCTCAAG
	try7-R	GCTGGCTTAGAGTTACCCCA
5.320	try9-F	CTTCTGTGGAAACTCGCTGG
	try9-R	ACAAACACCAATGGAAGCCC
2.1634	trans4-F	GCACTGTTGAGCGAAAGGAA
	trans4-R	TGTTTCAGGTTGGTGGCATG
2.896	trans5-F	TCCTTGCTCGTTCCCTCATT
	trans5-R	AGTACATCCACGACTGCCAA

## Data Availability

The clean reads data of the genome were deposited into the Sequence Read Archive (SRA) database of NCBI (accession number: SRR27030883 and SRR27030884). The genome has been deposited into the BioProject database of NCBI (accession number: PRJNA1047957). The transcriptome data presented in this study are available on request from the corresponding author.

## Supplementary Materials

The supporting information can be downloaded at: https://www.mdpi.com/article/10.3390/ijms25041965/s1.
